# Three-dimensional heads-up pneumatic retinopexy with chandelier illumination for rhegmatogenous retinal detachment in eyes with mild media opacity: a prospective case series

**DOI:** 10.1007/s10792-025-03804-y

**Published:** 2025-10-23

**Authors:** Ruggero Tartaro, Alberto Quarta, Maria Ludovica Ruggeri, Andrea Colonna, Rodolfo Mastropasqua

**Affiliations:** 1 Department of Neurosciences, Imaging and Clinical Sciences, University “G. d’Annunzio” Chieti-Pescara, Chieti, Italy; 2 Azienda Sanitaria Locale Alessandria, Alessandra, Italy; 3 Department of Sciences, Ophthalmology Clinic, Gabriele D’Annunzio University, National Center of High Technology in Ophthalmology, Via Dei Vestini, 66100 Chieti, Italy

**Keywords:** Pneumatic retinopexy, 3D heads-up surgery, Chandelier, Retinal detachment

## Abstract

**Purpose:**

To investigate a new technique for Pneumatic Retinopexy (PnR) using a 25G chandelier, cryotherapy, SF6 gas injection, and a heads-up 3D visualization system for rhegmatogenous retinal detachment (RRD).

**Methods:**

This prospective consecutive case series included 20 eyes from 20 patients with RRD including 11 macula-on and 9 macula-off cases. The surgical technique combined endoillumination via a 25-gauge chandelier with three-dimensional heads-up visualization, cryotherapy, and pure SF_6_ gas as an intraocular tamponade. All patients underwent a 12-month postoperative follow-up to assess primary retinal reattachment success and document any procedure-related complications.

**Results:**

The mean operative time was 10.3 ± 2.4 min. Primary anatomical success defined as single-procedure retinal reattachment without additional surgery was achieved in 80% of eyes (16/20) at 12 months. Median time to recurrence was 14 days. Of the four eyes with recurrence, two underwent a second gas injection with cryotherapy, and two required pars plana vitrectomy with silicone oil tamponade for new inferior breaks associated with proliferative vitreoretinopathy. Best-corrected visual acuity (BCVA) improved significantly from 0.54 ± 0.34 to 0.32 ± 0.23 LogMAR at 12 months postoperatively (*p* < 0.05).

**Conclusion:**

3D heads-up PnR with chandelier illumination is a feasible technique that offers subjectively enhanced intraoperative visualization compared with indirect ophthalmoscopy, particularly in eyes with mild media opacity. Future studies should investigate differences in clinical outcomes and cost-effectiveness.

**Supplementary Information:**

The online version contains supplementary material available at 10.1007/s10792-025-03804-y.

## Introduction

Pneumatic retinopexy (PnR) is a surgical technique employed in the treatment of rhegmatogenous retinal detachment (RRD), demonstrating efficacy particularly in cases involving superior retinal breaks [1, 2]. The technique is indicated for eyes with one or more retinal breaks located within the superior 4–8 clock hours, without evidence of proliferative vitreoretinopathy (PVR) or significant media opacity. When performed with appropriate case selection, adequate preoperative examination and standard surgical technique, PnR achieves high anatomical success rates [3]. The standard procedure involves the intravitreal injection of a gas bubble which ascends to provide tamponade, thereby facilitating the reattachment of the retina to the underlying retinal pigment epithelium.

Although indirect ophthalmoscopy (IO) is a cost-effective and non-invasive tool for performing PnR, retinal breaks may occasionally be missed due to media opacities or poor visualization. In addition, the current generation of ophthalmologists are less familiar with IO, while recent advances in surgical technology provide opportunities to further refine retinal detachment repair, improving both precision and efficacy [4–6].

In this prospective consecutive surgical case series we investigated the use of PnR with the intraoperative use of a 25 Gauge chandelier combined with a 3D surgical system in eyes with media opacities. Accordingly, we evaluated the 3D heads-up approach for PnR in carefully selected eyes focusing on feasibility, safety, and surgeon-rated visualization quality relative to IO while acknowledging standard PnR remains first-line in appropriately selected cases [3]. Additionally, by assessing intraoperative and postoperative complications, the study aims to establish the safety profile of this technique, providing a comprehensive evaluation of its potential benefits and limitations in a clinical setting.

## Methods

This prospective, consecutive surgical case series included 20 eyes from 20 patients diagnosed for RRD enrolled at the Ophthalmology Clinic of University “G. D’Annunzio”, Chieti-Pescara, Italy, and Casale Monferrato Hospital between April-July 2024. The study adhered to the tenets of the Declaration of Helsinki, it received approval from the relevant institutional ethical committee, and all patients signed a written specific informed consent to participate.

Inclusion criteria were: (1) one or more retinal breaks located between the 10 and 2 o’clock meridians assessed with IO and confirmed intraoperatively, (2) age > 18 years, able to provide informed consent and (3) willing to comply with scheduled follow-up examinations over a 12-month postoperative period. Patients were excluded if retinal breaks were located outside the 10–2 o’clock meridian, or if they had giant retinal tears, PVR grade B or C or higher, or subtotal vitreous hemorrhage precluding adequate retinal visualization. Additional exclusion criteria included prior retinal surgery in the affected eye, ocular comorbidities likely to affect visual prognosis such as advanced glaucoma or diabetic retinopathy, and inability to attend the scheduled follow-up visits. Eyes with mild corneal, lenticular, or vitreous opacities that did not preclude identification of retinal breaks were included. Eyes with dense opacities that obscured the fundus view were excluded”.

All patients underwent a complete ophthalmologic examination, including best corrected visual acuity (BCVA) expressed in logarithm of the minimum angle of resolution (logMAR) for statistical analysis, intraocular pressure (IOP) evaluated with Goldman applanation tonometry, slit lamp biomicroscopy, axial length measurement (IOL Master 700, Zeiss Meditech), fundus examination with indirect ophthalmoscopy after 1% tropicamide instillation. Media opacity was evaluated preoperatively by slit-lamp biomicroscopy for anterior segment clarity and by dilated fundus examination for posterior segment visualization. In cases where the fundus view was partially obscured, the degree of opacity was further documented using IO and, when necessary, graded according to its impact on visualization of the retinal periphery. The severity of lens opacities was graded using the Lens Opacities Classification System III (LOCS III) [7], and eyes with nuclear opalescence or cortical opacity greater than grade 3, or posterior subcapsular opacity greater than grade 3, were excluded. Corneal opacities and vitreous opacities, including asteroid hyalosis or mild vitreous hemorrhage, were permitted if they did not obscure the central pupillary area or preclude identification of retinal breaks. Eyes with dense corneal edema, dense corneal leukoma, or vitreous hemorrhage severe enough to prevent retinal visualization were excluded from the study [8]. Each patient underwent thorough preoperative evaluations to confirm the anatomical and clinical characteristics of their detachment, including slit-lamp examination and swept-source optical coherence tomography (SS-OCT) (Topcon Triton, Topcon, Japan). A posterior vitreous detachment (PVD) was assessed by the observation of the Weiss’ ring.

## Outcomes

The primary outcome was single-procedure anatomical success, defined as complete retinal reattachment without recurrence at 12 months following the initial pneumatic retinopexy. Secondary outcomes included final anatomical success (reattachment at 12 months regardless of additional procedures), change in BCVA at 12 months, incidence and type of complications, need for reoperation, and time to recurrence of retinal detachment. Recurrence was defined as any new subretinal fluid involving the macula or extending ≥ 2 disc diameters from the nearest retinal break after initial reattachment. PVR was graded according to the updated Retina Society classification, with grade C defined as full-thickness retinal folds associated with fixed star folds or subretinal bands. The second gas injection was performed only in cases of recurrent detachment without PVR, in which the new break location was suitable for pneumatic repair.

## Surgical technique

Before each procedure, the operating surgeon sequentially evaluated both visualization modalities—IO with scleral indentation and 3D PnR—under comparable lighting and patient positioning conditions. Four visualization parameters were assessed for each modality by a single surgeon (RT): illumination, defined as the perceived brightness and uniformity of light within the observed retinal area; overall image quality, referring to the clarity, sharpness, and contrast sufficient to discern anatomical landmarks and subtle pathology; field of view width, representing the extent of retina visible at one time without repositioning the system; and magnification, indicating the apparent enlargement of retinal structures to facilitate detailed inspection of lesions or breaks. Each parameter was rated immediately after use of the modality on a standardized 5-point Likert scale, where 1 represented the poorest and 5 the best possible performance. Study-related activities such as visualization scoring were performed outside of this measurement and were not included in the operative time.

All procedures were performed under local anesthesia using a Sub-Tenon's block. A 3D heads-up visualization system (NGENUITY, Alcon) was employed, allowing the surgical team to operate with enhanced depth perception and ergonomic efficiency through high-resolution imaging displayed on a screen. The imaging preset mode 'Tissue Detail Mode' was selected as it provides enhanced contrast and tissue differentiation, allowing earlier visualization of cryotherapy orange-reaction and helping to avoid over-treatment of rhegmatogenous lesions (Video 1).

The procedure began with the insertion of a 25G chandelier (Constellation, Alcon) in the inferotemporal quadrant, which provided critical endo illumination that improved visualization of the retinal landscape. This step was followed by obtaining a wide-angle view of the retina using a non-contact system (BIOM, Oculus).

Retinal breaks were precisely located by indenting the sclera with a cryotherapy probe. Upon accurate localization, the breaks were treated with cryotherapy. Through the observation of a subtle orange-reaction of the treated area, we considered the cryotherapy treatment effective. All retinal breaks identified—whether within or beyond the detached area—were treated with cryotherapy to ensure complete closure. A 0.5 ml anterior chamber paracentesis was performed using a 25G needle to reduce the intraocular pressure. Following the paracentesis, 0.5 ml of pure SF6 gas was injected into the vitreous cavity. The use of the 3D heads-up system allowed direct visualization of the gas bubble formation and monitoring of the perfusion of the central retinal artery. Patients were instructed to maintain a prone position if the macula was on or a supine position if the macula was off for the first 2 h following gas injection. Thereafter, positioning was adjusted relative to the location of the retinal break(s). In addition, in macula-off cases, the *steamroller technique* was applied, in which the patient’s head was positioned so that the expanding gas bubble gradually displaced subretinal fluid toward the peripheral break, promoting sequential retinal reattachment from the posterior pole toward the periphery.

## Statistical analysis

Descriptive statistics were calculated for all variables, with continuous data summarized as mean ± standard deviation (SD) or median with interquartile range (IQR), and categorical data expressed as counts and percentages. BCVA changes between preoperative and postoperative time points were assessed using the Wilcoxon signed-rank test.

Surgeon-assigned visualization scores for image quality, field of view, magnification, and illumination—collected on a 5-point Likert scale and paired within the same eyes—were compared between modalities using paired Wilcoxon signed-rank tests for each parameter. To account for multiple comparisons across the four parameters, *p*-values were adjusted using the Holm method. A total visualization score (sum of all four parameters) was also compared between modalities using the Wilcoxon signed-rank test; summation of Likert items was considered justified as they reflected distinct yet related aspects of visualization quality, and internal consistency was confirmed with Cronbach’s alpha. Statistical significance was set at *p* < 0.05 (two-tailed) after adjustment. Analyses were performed using R software (version 4.1; http://www.r-project.org/).

## Results

### Baseline characteristics

A total of 20 eyes from 20 patients with RRD underwent surgery, including 11 macula-on and 9 macula-off cases. The mean age was 61.5 years (range, 45–73 years); 12 patients (60%) were male and 8 (40%) were female. Ocular comorbidities causing media opacity are summarized in Table 1. All retinal breaks were located between the 10 and 2 o’clock meridians, in accordance with the inclusion criteria. PVD was confirmed in all cases. The mean operative time was 10.3 ± 2.4 min. All retinal breaks causing the detachment were located between the 10 and 2 o’clock meridians. Break morphology consisted of flap-type tears in all cases, with sizes ranging from 0.5 to 2 disc diameters. No giant retinal tears or dialyses were observed.

**Table 1:** Baseline demographic, ocular, and clinical characteristics of the study cohort

#	Age (Years)	Gender	Pre-op BCVA (LogMAR)	Type of media opacity	Lens status	Axial length (mm)	Tobacco dust visible	Number of tears in the detached area	Number of tears in the not detached area	Macula on or off	Reattachment with only the first PnR (Yes/No)	Post-op visual acuity (LogMAR)
1	56	Male	0,3	Corneal leukoma	Phakic	25,15	No	1	0	On	Yes	0,1
2	68	Female	0,9	Vitreous haemorrhage	Pseudophakic	24,83	No	2	0	Off	Yes	0,2
3	45	Male	0,9	Vitreous haemorrhage	Phakic	25,22	Yes	1	0	Off	No	0,8
4	72	Female	0,1	Corneal leukoma	Pseudophakic	25,66	Yes	3	1	On	Yes	0,3
5	59	Male	0,9	Corneal oedema	Phakic	24,78	No	1	0	Off	Yes	0,2
6	64	Female	0,6	Cataract N3C3P1	Pseudophakic	24,78	Yes	2	1	On	Yes	0,3
7	50	Male	0,9	Cataract N2C2P0	Phakic	25,69	No	1	0	Off	No	0,8
8	73	Female	0,3	Hyalosis asteroid	Pseudophakic	25,28	Yes	4	2	On	Yes	0,4
9	61	Male	0,4	Hyalosis asteroid	Phakic	24,67	No	2	1	On	Yes	0,2
10	67	Female	0,9	Corneal oedema	Pseudophakic	25,17	Yes	1	0	Off	Yes	0,1
11	68	Male	0,3	Cataract N2C2P2	Phakic	26,68	No	1	0	On	No	0,8
12	72	Female	0,9	Cataract N3C3P1	Pseudophakic	28,2	Yes	2	0	Off	No	0,4
13	74	Male	0,3	Cataract N2C2P0	Phakic	29,14	No	1	2	On	Yes	0,2
14	75	Female	0,1	Cataract N2C2P2	Pseudophakic	21,19	No	1	0	On	Yes	0,4
15	78	Male	0,9	Cataract N3C3P1	Pseudophakic	23,23	Yes	1	0	Off	Yes	0,2
16	88	Male	0,9	Cataract N2C2P2	Phakic	23,8	No	1	0	Off	Yes	0,4
17	87	Male	0,1	Cataract N3C3P3	Phakic	24,48	Yes	1	0	On	Yes	0,2
18	65	Female	0,9	Cataract N2C2P0	Pseudophakic	26,27	No	2	0	Off	Yes	0,1
19	56	Male	0,1	Cataract N3C3P1	Phakic	22,2	Yes	2	0	On	Yes	0,2
20	67	Female	0,1	Cataract N2C2P3	Phakic	26,21	No	1	0	On	Yes	0,1

The table summarizes age, gender, preoperative best-corrected visual acuity (BCVA, LogMAR), type of media opacity, lens status, axial length, posterior vitreous detachment status, presence of tobacco dust, number and distribution of retinal tears, macular status at presentation, reattachment with the first pneumatic retinopexy (PnR) alone, and postoperative BCVA at final follow-up. Media opacities include corneal leukoma, corneal edema, cataract graded using the Lens Opacities Classification System III (LOCS III), asteroid hyalosis, and vitreous hemorrhage

### Anatomical and functional outcomes

Primary anatomical success (single-procedure) was achieved in 16 of 20 eyes (80%) at 12 months. Four eyes (20%) experienced RD recurrence after the initial procedure. The median time to recurrence was 14 days (IQR, 10–21 days). Of these, two eyes (cases 3 and 7) developed new inferior retinal breaks located between 5 and 7 o’clock, while the remaining two (cases 11 and 12) developed recurrences without new inferior breaks. No sclerotomy-related breaks were identified at the chandelier entry sites in any case. All peripheral breaks causing the detachment were identified intraoperatively and were treated, including 3 breaks in 3 eyes outside the detached retina localized over flat areas. No significant intraoperative complications were observed. BCVA improved significantly from a preoperative mean of 0.54 ± 0.34–0.32 ± 0.23 LogMAR at 12 months postoperatively (*p* < 0.05).

### Comparative assessment of visualization techniques

When comparing the four visualization parameters between 3D PnR and IO, higher mean scores were observed with 3D PnR for image quality (4.75 vs 3.75; + 1.00), field of view (4.51 vs 3.71; + 0.80), magnification (4.76 vs 3.25; + 1.51), and illumination (4.71 vs 3.72; + 0.99). After adjustment for multiple comparisons using the Holm method, the total visualization score—representing the sum of all four parameters—remained significantly higher for 3D PnR (18.73 vs 14.43; + 4.30; *p* = 0.006). Internal consistency for the combined score was high (Cronbach’s α > 0.9), supporting the summation of Likert-scale items (Table 2).

**Table 2:** Mean surgeon-assigned scores for visualization parameters when performing pneumatic retinopexy using a 3D heads-up display with chandelier illumination (3D PnR) versus conventional indirect ophthalmoscopy (IO)

Parameter	3D PnR (mean)	IO (mean)	Difference
Image quality	4.75	3.75	+ 1.00
Field of view	4.51	3.71	+ 0.80
Magnification	4.76	3.25	+ 1.51
Illumination	4.71	3.72	+ 0.99
Total score	18.73	14.43	+ 4.30

Scores were assigned on a 5-point Likert scale, with higher values indicating better perceived performance. Across all parameters—image quality, field of view, magnification, and illumination—3D PnR received higher mean ratings, resulting in a greater total visualization score. Differences reflect paired comparisons within the same eyes

## Discussion

In this prospective and consecutive surgical case series we demonstrated that 3D PnR is associated with high anatomical success and significant postoperative visual improvement in selected cases of RRD. This is the second study investigating the use of a 25G chandelier in PnR, following the foundational work by Habib et al. [9], with our work that incorporates further advances in this approach by introducing the 3D heads-up visualization system.

Retinal reattachment achieved a success rate of 80% after the initial procedure, similarly to the standard PnR technique [3, 10–12]. Following the initial procedure, two patients required additional interventions: one underwent a second gas injection, and another required vitrectomy with oil tamponade due to new PVR and inferior breaks. These findings fall within the reported range of success rates for PnR, which vary between 60 and 91% depending on patient selection criteria. The relatively high success rate achieved in this study may also be attributed to the careful selection of patients, especially the superior location of the breaks and the PVD. The mean operative time was 10.3 ± 2.4 min, comparable to conventional PnR [1, 2].

We did not find any postoperative aniseikonia, which could be a complication of PnR [13]. The current technique provides as major advantages the ability to directly visualize the central retinal artery while the gas is injected. This allows for real-time monitoring and adjusting of perfusion, ensuring adequate central retinal artery blood flow. Additionally, the direct visualization during gas injection prevents the formation of "fish eggs" or small gas bubbles within the vitreous cavity because it is possible to titrate the gas injection speed and the needle positioning. A direct visualization of the needle can help position the needle close to the eyewall, avoiding forming fish eggs, thus creating a better tamponade effect [2].

We used cryotherapy instead of laser coagulation because the latter can be technically challenging in the presence of subretinal fluid. On the other hand, cryotherapy works well even if the retina is detached and reduces the need for postoperative laser photocoagulation.

We used SF6 which provides a balanced duration of tamponade since in PnR excessive or insufficient tamponade can determine the retinal reattachment [14]. The selection of SF6 mitigates the risks of under- and over-tamponade while maintaining a favorable safety profile. Despite the benefits offered by the 3D heads-up visualization technology in PnR, these advantages must be balanced against increased costs and a more invasive procedure (due to the 25G chandelier), compared to traditional methods.

The prospective case series demonstrates the feasibility of performing PnR using a 3D heads-up display combined with chandelier illumination. The approach provided the operating surgeon with subjectively enhanced visualization—particularly in terms of image quality, magnification, field of view, and illumination—compared with conventional IO. However, the study was not designed or powered to detect differences in anatomical success rates or other clinical outcomes relative to standard PnR. The primary intent was to evaluate technical feasibility and potential visualization advantages in selected eyes, particularly in those with mild media opacity. While the 3D display does not bypass optical opacity, when combined with chandelier endoillumination and BIOM wide-angle optics it provided subjectively enhanced contrast and magnification compared with IO. Another limitation of this study is that the visualization parameters were rated by the operating surgeon, who was aware of which modality was being assessed. This awareness may introduce observer bias. Although identical examination conditions and a standardized scoring rubric were used to reduce variability, future studies could incorporate masked (blinded) evaluators or video-based image assessments by independent graders to further minimize subjective bias and improve reproducibility of the results. Although chandelier insertion theoretically introduces an additional risk of infection, no cases of endophthalmitis or wound-related complications were observed in our 20 cases. The use of a single 25G self-sealing entry site and careful aseptic technique likely minimized this risk, consistent with prior chandelier-assisted procedures [15].

While the 25G chandelier may not currently represent the most cost-effective choice, our work reveals its potential to enhance surgical efficiency. Future studies designed to include cost-effectiveness analyses may be useful. The enhanced visualization and ergonomic benefits of the 3D heads-up system and chandelier setup may justify the increased costs in specific scenarios. However, several limitations must be acknowledged to guide future research. First, the relatively small sample size limits the study's statistical power. Second, potential selection bias must be evidenced, as the study exclusively included cases with superior retinal tears between 10 and 2 o'clock, generally associated with more favorable outcomes in the case of PnR. As a result, the findings may not directly apply to a broader spectrum of retinal detachment cases, where outcomes might differ significantly.

In conclusion, a combined technique seems to be promising in elevating the visualization in eyes with opaque media undergoing PnR. Still, future studies are needed to evaluate the usefulness and cost-effectiveness in clinical practice. Our results align with other research emphasising the need for a thoughtful integration of traditional and advanced technologies to improve surgical outcomes.

## Supplementary Information

Below is the link to the electronic supplementary material.

Supplementary file1 (MP4 73334 KB)

## Data Availability

Data and results supporting this study’s findings are available upon reasonable request to the corresponding author.

## References

[1] Stewart S, Chan W (2018) Pneumatic retinopexy: patient selection and specific factors. Clin Ophthalmol Volume 12:493–502. 10.2147/OPTH.S137607 10.2147/OPTH.S137607 PMC5859893 29588570

[2] Huang CY, Mikowski M, Wu L (2022) Pneumatic retinopexy: an update. Graefes Arch Clin Exp Ophthalmol 260(3):711–722. 10.1007/s00417-021-05448-x 34636994 10.1007/s00417-021-05448-x

[3] Hillier RJ, Felfeli T, Berger AR et al (2019) The pneumatic retinopexy versus vitrectomy for the management of primary rhegmatogenous retinal detachment outcomes randomized trial (PIVOT). Ophthalmology 126(4):531–539. 10.1016/j.ophtha.2018.11.014 30468761 10.1016/j.ophtha.2018.11.014

[4] Mastropasqua R, Quarta A, Ruggeri ML, Mastropasqua L (2025) Enhancing precision and clarity with new digital color assistant in 3D heads-up vitreoretinal surgery. Ophthalmol Ther 14(4):805–814. 10.1007/s40123-025-01106-1 39962031 10.1007/s40123-025-01106-1 PMC11920492

[5] Mastropasqua R, Ruggeri ML, Quarta A et al (2025) New mixed reality headset: first exploratory use in intraocular surgery and telementoring. Ophthalmol Ther 14(7):1597–1609. 10.1007/s40123-025-01117-y 40214829 10.1007/s40123-025-01117-y PMC12167422

[6] Mudvari SS, Ravage ZB, Rezaei KA (2009) Retinal detachment after primary pneumatic retinopexy. Retina 29(10):1474–1478. 10.1097/IAE.0b013e3181ae70f3 19730164 10.1097/IAE.0b013e3181ae70f3

[7] Chylack LT (1993) The lens opacities classification system III. Arch Ophthalmol 111(6):831. 10.1001/archopht.1993.01090060119035 8512486 10.1001/archopht.1993.01090060119035

[8] Standardization of Uveitis Nomenclature for Reporting Clinical Data (2005) Results of the first international workshop. Am J Ophthalmol 140(3):509–516. 10.1016/j.ajo.2005.03.057 16196117 10.1016/j.ajo.2005.03.057 PMC8935739

[9] Habib AE, Abdel-Kader AA, Elnahry AG (2021) Chandelier-assisted pneumatic retinopexy for rhegmatogenous retinal detachment repair in young adults. Indian J Ophthalmol 69(4):979–981. 10.4103/ijo.IJO_1798_20 33727471 10.4103/ijo.IJO_1798_20 PMC8012970

[10] Modi YS, Epstein A, Flynn HW, Shi W, Smiddy WE (2014) Outcomes and complications of pneumatic retinopexy over a 12-year period. Ophthalmic Surg Lasers Imaging Retina 45(2):132–137. 10.3928/23258160-20140306-06 24635154 10.3928/23258160-20140306-06

[11] Rootman DB, Luu S, Conti SM et al (2013) Predictors of treatment failure for pneumatic retinopexy. Can J Ophthalmol 48(6):549–552. 10.1016/j.jcjo.2013.05.002 24314421 10.1016/j.jcjo.2013.05.002

[12] İpekli Z, Pehlivanoğlu S, Artunay Ö (2023) Efficacy of pneumatic retinopexy in young adults with rhegmatogenous retinal detachment. Ther Adv Ophthalmol 15:25158414231208280. 10.1177/25158414231208279 37915881 10.1177/25158414231208279 PMC10617287

[13] Griffin S, Chan L, McCarthy K et al (2025) Pneumatic retinopexy for rhegmatogenous retinal detachment outcomes. Ophthalmol Retina 9(5):437–443. 10.1016/j.oret.2024.10.025 39521131 10.1016/j.oret.2024.10.025

[14] Yeung L, Kokame GT, Brod RD, Lightman DA, Lai JC (2011) Pneumatic retinopexy for retinal detachment associated with severe choroidal detachment. Retina 31(1):87–92. 10.1097/IAE.0b013e3181e0974c 20714274 10.1097/IAE.0b013e3181e0974c

[15] Kao YS, Chen CY, Huang YT, Chen SN (2024) Comparison of chandelier-assisted versus standard scleral buckling for the treatment of primary rhegmatogenous retinal detachment: a systematic review and meta-analysis. Ophthalmologica 247(5–6):345–354. 10.1159/000540820 39159609 10.1159/000540820 PMC11614423

